# Efficacy and Safety of Nivestim Versus Neupogen for Mobilization of Peripheral Blood Stem Cells for Autologous Stem Cell Transplantation

**DOI:** 10.1038/s41598-019-56477-w

**Published:** 2019-12-27

**Authors:** Cindy Chew, Hong Yen Ng

**Affiliations:** 1 Department of Pharmacy, National Cancer Centre, Singapore, 11 Hospital Crescent, 169610 Singapore; 2 Department of Pharmacy, Singapore General Hospital, Block 7 Level 2, Outram Road, 169608 Singapore

**Keywords:** Haematological cancer, Leukaemia

## Abstract

A retrospective cohort study was conducted in Singapore General Hospital to study the safety and efficacy of biosimilar granulocyte-colony stimulating factor (G-CSF) Nivestim for chemo-mobilization of stem cells for autologous stem cell transplant (autoSCT). All patients who underwent an autoSCT between January 2011 and December 2016 were screened for eligibility. A total of 194 patients were screened, and 131 were included. Nivestim was used in 65 patients and the originator G-CSF (Neupogen) in 66. Patient characteristics were similar between both arms except for chemo-mobilization regimen used (p < 0.0001). Mobilization success rates were found to be comparable, at 96.9% (Nivestim) and 97% (Neupogen). Adverse events rates were also similar. Median duration of G-CSF use and hospitalization were both found to be shorter in the Nivestim arm. Median drug acquisition cost per mobilization cycle was significantly lower in the Nivestim arm at $533.40 (range $213.40–$1280.20) as compared to $1261.90 (range $574–$2755.20) in the Neupogen arm (p < 0.0001). No difference was observed for neutrophil and platelet engraftment after autoSCT. Nivestim was found to be safe and non-inferior to Neupogen for chemo-mobilization of stem cells for autoSCT, and associated with lower cost and shorter length of hospitalization.

## Introduction

Autologous stem cell transplantation (autoSCT) is an important treatment modality in the management of various chemo-sensitive malignant conditions including multiple myeloma, Hodgkin’s and non-Hodgkin’s lymphoma, soft tissue sarcoma, germ cell cancer and Ewing sarcoma^1,2^. In the management of multiple myeloma, autoSCT is currently the standard of care, even in this era of novel agents^3^. A recent meta-analysis conducted by Dhakal *et al*. demonstrated superior progression free survival with autoSCT even with the use of novel agents during induction therapy^4^. In the setting of relapsed lymphoma, autoSCT can offer as an alternative management option if patients failed systemic treatment. The National Comprehensive Cancer Network (NCCN) guidelines have listed autoSCT as standard of care for lymphoma patients in second remission^5,6^. The Parma study demonstrated that salvage chemotherapy followed by autoSCT yielded a superior 5-year event free survival when compared to salvage chemotherapy alone in patients with relapsed lymphoma^7^.

Mobilization of peripheral blood stem cells (PBSCs) upon achieving remission after induction chemotherapy constitutes a critical component in the continuum of an autoSCT. These PBSCs are then frozen and stored for reinfusion into the patient as a form of “rescue” after administering high dose chemotherapy, constituting an autoSCT. Recombinant granulocyte colony-stimulating factor (G-CSF) plays a pivotal role in ensuring success of an autoSCT. It is required to mobilize stem cells from the bone marrow into the peripheral blood to allow for harvesting via apheresis. Chemotherapy may be employed to facilitate the process of mobilization by causing a rebound production of stem cells in the bone marrow for harvesting (chemo-mobilization). Besides, G-CSF is also essential in accelerating hematopoietic recovery after autoSCT^8–10^.

With the recent expiry of patent for Neupogen, many biosimilar G-CSFs have been developed and made available. A biosimilar is a biological agent that is comparable in terms of quality, safety and efficacy to the approved original biological medicine^11,12^. These biosimilars were developed with the intent of reducing cost as compared to the originator, translating to more affordable healthcare. The manufacturing processes of these biosimilars were independent of the proprietary methods. Hence, the quality, purity and clinical activity of biosimilars may be of concern^11^.

Biosimilar G-CSFs have been shown to exhibit bioequivalence to Neupogen in terms of pharmacokinetics and pharmacodynamics properties^8,13,14^. In a meta-analysis of three large randomized, two-arm study for the management of chemotherapy-related neutropenia in non-Hodgkin’s lymphoma, breast and lung cancers, biosimilar G-CSFs were found to be comparable to Neupogen^9^. Based on the available data, biosimilar G-CSFs were approved by the European Medicines Agency (EMA) in 2010 for the same indications as Neupogen based on comparable efficacy, quality and safety^10,15^. However, the long term safety data as well as effects on quality of PBSCs are still lacking.

In April 2014, a biosimilar G-CSF, Nivestim was introduced into Singapore General Hospital (SGH) to be used in place of Neupogen, with the exception of mobilization of PBSCs for allogeneic stem cell transplant due to the unknown long-term effect on healthy donors. While biosimilar G-CSFs are approved for mobilization of PBSCs for autologous HSCT, majority of the studies examining the efficacy and safety in this setting involved biosimilar, Zarzio. There were concerns whether the different manufacturing processes will result in micro-heterogeneity among the biosimilars, affecting the mobilization process and quality of PBSCs mobilized for transplant^8^. With limited evidence on the effect of Nivestim for mobilization of stem cells and resultant recovery from an autoSCT, this study aimed to compare the efficacy and safety of biosimilar G-CSF (Nivestim) with originator G-CSF (Neupogen) in the context of chemo-mobilization.

## Method

### Study design

This was a single center, retrospective cohort study conducted at SGH, comparing the efficacy and safety of biosimilar G-CSF (Nivestim) with originator G-CSF (Neupogen) in chemo-mobilization of PBSCs for autoSCT. This study was approved by Singhealth Centralized Institutional Review Board (CIRB Ref 2016/2233) with waiver of informed consent.

All patients who underwent autoSCT in SGH from January 2011 to November 2016 were screened for eligibility. Patients who underwent autoSCT from January 2011 to March 2014 received Neupogen for mobilization while the others received Nivestim due to change in the hospital drug formulary in April 2014.

### Study participants

Inclusion criteria included at least 18 years of age; diagnosis of multiple myeloma, lymphoma or leukemia; and underwent first chemo-mobilization for autoSCT. Patients were excluded if they were diagnosed with any other autoimmune or hematological disease apart from the above-mentioned conditions; previously failed mobilization; received both plerixafor and G-CSF, or Pegylated G-CSF for mobilization; or had prior transplant.

### Sample size calculation

Sample size was calculated using an 80% power and a two-sided α = 5% to detect a non-inferiority of 20% between Neupogen and Nivestim. This yielded a sample size of 65 patients per arm.

### Outcomes

The primary outcome was the proportion of mobilization success, defined as a harvest of at least 2 × 10^6^ CD34^+^ cells per kilogram body weight. Secondary outcomes included incidence and severity of adverse events, total number of cells mobilized, duration of injections received per mobilization, number of G-CSF injections received, number of days of apheresis required, length of hospitalization for mobilization, drug acquisition cost of G-CSF, and time to engraftment of neutrophil and platelet after subsequent autoSCT. Severity of adverse events was graded based on Common Terminology Criteria for Adverse Events (CTCAE) version 4.03. Length of hospitalization for mobilization was calculated from the day of admission for planned PBSCs mobilization to discharge after completion of harvest. Time to engraftment of neutrophil and platelet was defined as the time taken for engraftment to take place after high dose chemotherapy and infusion of PBSCs. Neutrophil engraftment was defined as the first of the 3 consecutive days, on which the absolute neutrophil count (ANC) was 0.5 × 10^9^/L or greater, while platelet engraftment was defined as first of 3 consecutive days, on which the platelet count was 20 × 10^9^/L or greater without platelet transfusion in the preceding 7 days^16,17^.

### Statistical analysis

Categorical data including proportion of mobilization success and adverse events in both arms were compared using chi-square test. Continuous variables such as number of cells mobilized, duration and number of G-CSFs received, number of days of apheresis, length of hospitalization, and time to engraftment of neutrophil and platelet between the two arms were compared using Mann-Whitney U test. Binary logistic regression was performed to investigate potential confounding effects of parameters that were significantly different between the two arms at baseline. All other outcomes were reported using descriptive statistics. All statistical analyses were performed using IBM SPSS software version 24 and Microsoft Excel 2013.

## Results

### Patient demographics

**Figure 1:**
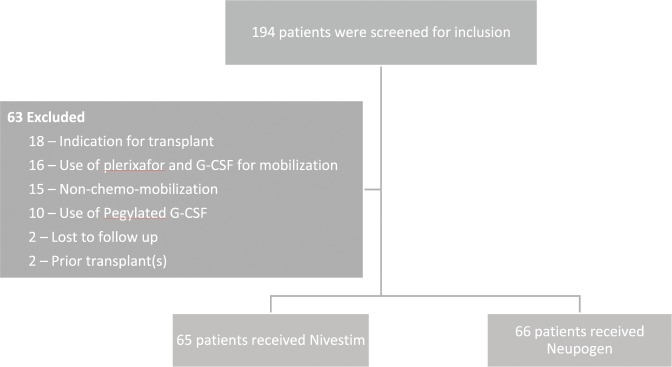
Patient recruitment.

A total of 194 patients who underwent autologous stem cell transplant from January 2011 to November 2016 were screened for inclusion. Among these, 63 patients were excluded due to various reasons as shown in Fig. 1. Of the patients included, 65 received Nivestim and 66 received Neupogen. Majority of the patients had multiple myeloma (n = 79), and were in complete remission or partial remission at transplant (n = 85). As shown in Table 1, baseline demographics were similar in both arms with the exception of the type of chemo-mobilization regimen used.

**Table 1:** Patient Demographics.

	Nivestim (N = 65)	Neupogen (N = 66)	p-value
**Gender, n (%)**			0.183
• Male	38 (58.5)	41 (62.1)	
**Median age (years)**	57	56	0.142
*Range*	20–71	18–73	
**Diagnosis, n (%)**			0.064
• Multiple Myeloma	34 (52.3)	45 (68.2)	
• Lymphoma	31 (47.7)	21 (31.8)	
**Median weight (kg)**	59.1	65	0.218
*Range*	39–94.7	38.2–138	
**Disease status, n (%)**			0.886
• Complete remission	29 (44.6)	33 (50)	
• Partial response	10 (15.4)	13 (19.7)	
• Progression	1 (1.5)	1 (1.5)	
• Relapse	25 (38.5)	19 (28.8)	
**Number of lines of treatment prior to mobilization, n (%)**			0.936
• 1	39 (60)	43 (65.2)	
• 2	24 (36.9)	17 (25.7)	
• ≥3	2 (3.1)	6 (9.1)	
**Chemo-mobilization regimen, n (%)**			<0.0001
• Cyclophosphamide/vinorelbine	27 (41.5)	22 (33.3)	
• High dose cyclophosphamide	6 (9.2)	25 (37.9)	
• Others	32 (49.2)	19 (28.8)	

### Mobilization success

Mobilization success was found to be similar, observed in 63 patients (96.9%) in the Nivestim arm as compared to 64 patients (97%) in the Neupogen arm (p = 0.988), as shown in Table 2. Following mobilization and harvesting, a higher number of patients proceeded on to autoSCT in the Nivestim arm (n = 56, 86.2%) as compared to the Neupogen arm (n = 49, 74.3%). Majority of the patients who did not proceed to receive autologous stem cell transplant were due to disease relapse, complications, or  expired before transplant. However, this difference did not reach statistical significance.

**Table 2:** Study Outcomes.

	Nivestim (N = 65)	Neupogen (N = 66)	p-value
**Primary Outcome**			
• Mobilization success, n (%)	63 (96.9)	64 (97)	0.988
**Secondary Outcomes**			
**Safety**			
• Incidence of adverse events, n (%)	12 (18.5)	5 (7.6)	0.583
*Back pain*	6*	4	
*Bone pain*	3*	1	
*Body aches*	2	0	
*Headaches*	1	0	
*Hip pain*	1	0	
*Atypical chest pain*	1	0	
• Missing data, n (%)	25 (38.5)	51 (77.3)	
**Mobilization and Apheresis**			
• Median number of cells mobilized (x 10^6^)	7.56	6.81	0.318
*Range*	0–45.9	0.2–40.63	
• Median duration of G-CSF received per mobilization (days)	9	10	0.126
*Range*	3–18	5–24	
• Median number of Injections received per mobilization	20	22	0.49
*Range*	8–48	10–48	
• Median number of apheresis sessions (days)	2	2	0.874
*Range*	0–4	1–7	
• Medium length of hospitalization (days)	15	17	0.035
*Range*	2–152	5–61	
Drug Acquisition Cost^†^			
• Median drug acquisition cost for G-CSF per patient ($)	533.4	1261.9	<0.0001
*Range*	213.4–1280.2	574–2755.2	
**Engraftment Kinetics**			
• Median time to neutrophil engraftment (days)	11	11	0.719
*Range*	9–16	9–17	
• Median time to platelet engraftment (days)	13	11.5	0.100
*Range*	9–26	8–22	

*2 patients in the Nivestim arm had both back pain and bone pain.

^†^Drug acquisition cost was calculated based on the drug cost at the time of the study.

Across both arms, majority of the patients received either cyclophosphamide 1.5 g/m^2^/vinorelbine 25 mg/m^2^ or single agent cyclophosphamide 4 g/m^2^ as chemo-mobilization regimen. Though the type of chemo-mobilization regimen used was significantly different between the two arms at baseline, analysis via logistic regression did not identify this as a potential confounder to our results. Sub-group analysis based on chemo-mobilization regimen yielded comparable success rate using either cyclophosphamide/vinorelbine or high dose cyclophosphamide regimen (Table 3). Similarly, sub-group analysis based on prior treatment lines did not reveal any statistical difference in mobilization success rates between patients with only one line of prior treatment and those who were more heavily pre-treated.

**Table 3:** Mobilization Success Sub-Group Analysis.

	Nivestim (N = 65)	Neupogen (N = 66)
**Chemo-mobilization regimen**		
• Cyclophosphamide/vinorelbine, n	27	22
*Mobilization success, n (%)*	26 (96.3)	21 (95.5)
• High dose cyclophosphamide, n	6	25
*Mobilization success, n (%)*	6 (100)	25 (100)
• Others, n	32	19
*Mobilization success, n (%)*	31 (96.9)	18 (94.7)
**Lines of treatment**		
• 1, n	39	43
*Mobilization success, n (%)*	37 (94.9)	43 (100)
• >1 line, n	26	23
*Mobilization success, n (%)*	26 (100)	21 (91.3)

*All p-value were >0.05.

### Safety

Safety profile of the two G-CSFs appeared to be similar. Incidence and severity of adverse events were comparable between Nivestim and Neupogen. However, a significantly higher proportion of patients in the Neupogen arm had missing safety data, as shown in Table 2. Headache, back pain, and bone pain were among the most commonly reported adverse events in both arms. The adverse events were mainly mild to moderate in severity, with none of the patients experiencing grade 3 and above events. There were also no cases of mortality.

### Mobilization and apheresis outcomes

The median number of CD34+ cells mobilized using Nivestim was higher at 7.56 × 10^6^ per kilogram body weight (range; 0 to 45.9) as compared to 6.81 × 10^6^ per kilogram body weight (range; 0.2 to 40.63) using Neupogen. However, this difference did not reach statistical significance. As shown in Table 2, the median number of apheresis days required was similar between the two groups; while the median length of hospitalization required for mobilization was significantly shorter for the Nivestim arm (p = 0.035). Two and six patients from the Nivestim and the Neupogen arm, respectively, had prolonged hospitalization beyond 30 days. Majority of the prolonged hospitalization cases were associated with severe sepsis, including an isolated case in the Nivestim arm where intensive care admission was required.

### Drug acquisition cost

The use of Nivestim for mobilization resulted in a significantly lower drug acquisition cost as compared to Neupogen, $533.4 vs $1261.9 (p < 0.0001), as indicated in Table 2. Of note, the median duration of G-CSF use per mobilization was lower when using Nivestim (9 days) as compared to that of Neupogen (10 days).

### Engraftment outcomes

As shown in Table 2, engraftment kinetics after subsequent autoSCT for both neutrophils and platelets were similar between the two groups.

## Discussion

Successful mobilization of sufficient PBSCs is of paramount importance in an autoSCT. It has primarily replaced stem cell harvest from the bone marrow due to ease of collection, avoidance of risk of general anaesthesia, as well as more rapid recovery of blood counts.

Several existing publications have demonstrated comparable safety and efficacy of biosimilar G-CSF with the originator in the context of PBSC mobilization for autoSCT. However, the bulk of these studies were not powered with sample size calculation, descriptive in nature, and mostly involved biosimilar of a different brand. This appropriately powered study, with the largest sample size to our knowledge so far, contributes to the growing pool of evidence supporting the use of biosimilar G-CSF.

Our data demonstrated comparable mobilization success rates between Nivestim (96.9%) and Neupogen (97%) after chemo-mobilization of PBSCs. These results were in concordance with other studies conducted in similar settings comparing biosimilar with originator G-CSF in chemo-mobilization.

In a retrospective study by Pham *et al*.^18^ in 98 multiple myeloma patients, a higher proportion of patients achieved mobilization success with the use of Nivestim (85%) as compared to Neupogen (81.5%), but no statistical significance (p = 0.78) was found. Though the same biosimilar was used, it is noteworthy that this study had a smaller sample size, and the patients included were less heavily pre-treated with only one line of induction therapy prior to mobilization.

In a study by Lefrère *et al*.^19^ in France, where the efficacy of biosimilar Zarzio was compared to Neupogen for chemo-mobilization in a population consisting of both multiple myeloma and lymphoma patients, it was found that the median CD34+ cells collected was comparable between both arms (p = 0.26), with a slightly higher number of cells collected from Zarzio (5.5 × 10^6^) as compared to Neupogen (4.49 × 10^6^). Another single-centred study conducted in Hungary by Reményi *et al*.^16^ also demonstrated a mobilization success rate of 91% for biosimilar Zarzio, comparable with published data for Neupogen.

There were no remarkable findings in terms of safety profile of Nivestim. Majority of the adverse events reported were expected and commonly encountered for G-CSF, and were mild to moderate in severity^20^. The commonly documented adverse events from the current study seemed comparable to other similar studies including the one by Lefrère *et al*., where 14 cases of bone pain and/or headache were noted among the 40 patients who received Zarzio, but no statistical analysis was performed^19^.

In terms of the apheresis process, the use of biosimilar Nivestim did not result in increased G-CSF injections or apheresis sessions required as compared to Neupogen.

Engraftment kinetics after reinfusion of PBSCs upon completion of high dose chemotherapy in autoSCT was included in the analysis as a surrogate marker of quality of the harvested PBSCs. The current study did not show any difference in time to engraftment between the Nivestim and Neupogen arms. From this finding, it appeared that the use of Nivestim did not affect quality of PBSCs mobilized, translating to comparable efficacy in stem cell mobilization.

While the current study looked into the drug acquisition cost of Neupogen and Nivestim for the mobilization process of an autologous stem cell transplant, it was not known how the use of biosimilars affected other indirect costs such as length and cost of hospitalization, number of injections and apheresis sessions required, and the apheresis cost incurred. A study by Severson C. also concluded that biosimilar was more affordable which  improved the access of G-CSF for use in stem cell transplant as per recommendation in guidelines^21^. Hence, to determine the true pharmacoeconomic outcome, a cost-minimization study may be considered.

### Limitations

The main limitation of this study was the retrospective nature, and resultant inability to match patients in the two arms based on patient demographics and chemo-mobilization regimen while maintaining the calculated sample size. In addition, data on adverse effects collected was dependent on the accuracy, consistency, and completeness of documentation in the clinical progress notes. There was also a high incidence of missing data on adverse events especially in the Neupogen arm due to unavailability of clinical progress notes. Lastly, analysis of both drug acquisition cost and other indirect costs would have provided a more comprehensive and meaningful pharmacoeconomic evaluation on the use of biosimilars in this setting.

## Conclusion

In conclusion, Nivestim was found to be safe and non-inferior to Neupogen in the chemo-mobilization of stem cells for autologous stem cell transplant, yet at a lower drug cost and a potentially shorter length of hospitalization.
